# Gastric Trichobezoar: An Unusual Cause of Anemia

**DOI:** 10.7759/cureus.79410

**Published:** 2025-02-21

**Authors:** Youssef Ghaddou, Zakaria Nsiri, Abdelaziz Fadil, Khalid Sair

**Affiliations:** 1 Surgery, Mohammed VI University of Sciences and Health - UM6SS, Casablanca, MAR

**Keywords:** adolescent, anemia, gastrotomy, psychiatry, trichobezoar

## Abstract

Trichobezoar is a rare disorder that causes swallowed hair to accumulate in the gastrointestinal tract. It can lead to various gastrointestinal and systemic complications. We report the case of a 16-year-old girl who presented with pallor, chronic lethargy, and unintended weight loss. Initial assessment revealed severe microcytic anemia unresponsive to conventional iron supplementation. A giant trichobezoar was found to be filling the stomach after additional imaging investigation. Previously undetected trichotillomania and trichophagia were present in the patient's history. After the trichobezoar was surgically removed, her anemia and general health significantly improved with multidisciplinary care that included behavioral therapy, psychiatric treatment, and nutritional support. This case emphasizes how crucial it is to include trichobezoar as a differential diagnosis in young adolescents who have unexplained anemia, especially when behavioral or psychological issues are present. Early recognition and multidisciplinary management are crucial to prevent severe complications.

## Introduction

A bezoar is a compact mass formed by indigestible materials that accumulate in the gastrointestinal tract. It is most frequently found in the stomach, as the pylorus serves as a natural barrier, preventing their further passage. Bezoars are categorized based on their composition into trichobezoars (masses of hair or hair-like fibers), phytobezoars (composed of vegetable or fruit fibers), pharmacobezoars (resulting from pills), lactobezoars (formed from milk and curd), and lithobezoars (made up of stone fragments) [1]. A gastrointestinal trichobezoar is a dense, solid accumulation of swallowed hair, accounting for 6% of all bezoar types. This condition predominantly affects young females, with 90% of cases occurring in individuals aged 13 to 20 years who exhibit tendencies for hair pulling (trichotillomania) and hair ingesting (trichophagia) [2]. This case report delves into the management challenges posed by a rare condition - trichobezoar presenting with iron deficiency anemia. This uncommon clinical presentation highlights the intricacies involved in reaching an accurate diagnosis, formulating appropriate treatment strategies, and addressing the broader implications for the patient's overall health and quality of life. By exploring this unique scenario, the report underscores the importance of a multidisciplinary approach in the management of this rare disease and its systemic manifestations.

## Case presentation

**Figure 1:**
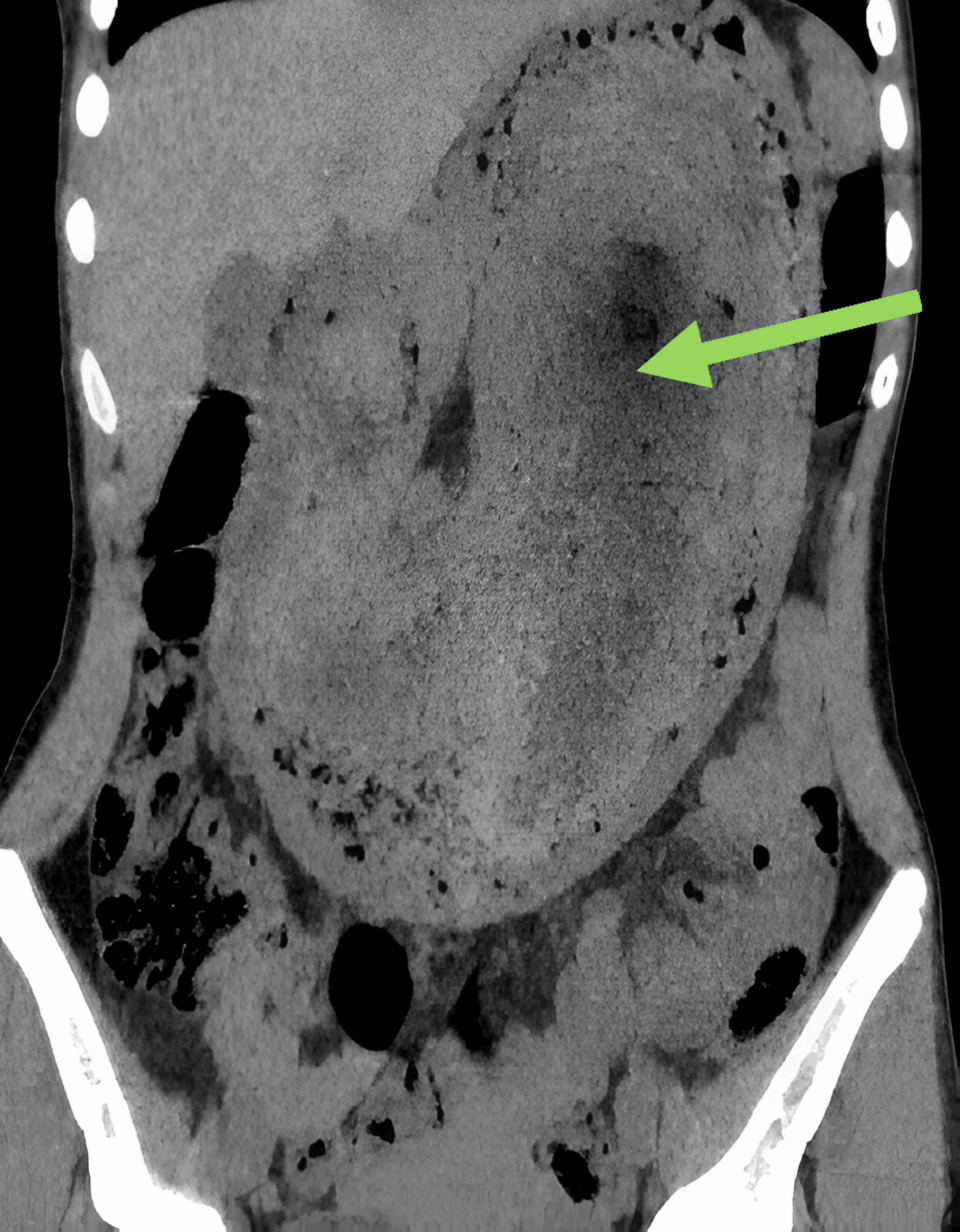
Abdomino-pelvic CT (coronal section) showing a diffuse, heterogeneous intragastric mass containing air and entrapped food debris (arrow).

**Figure 2:**
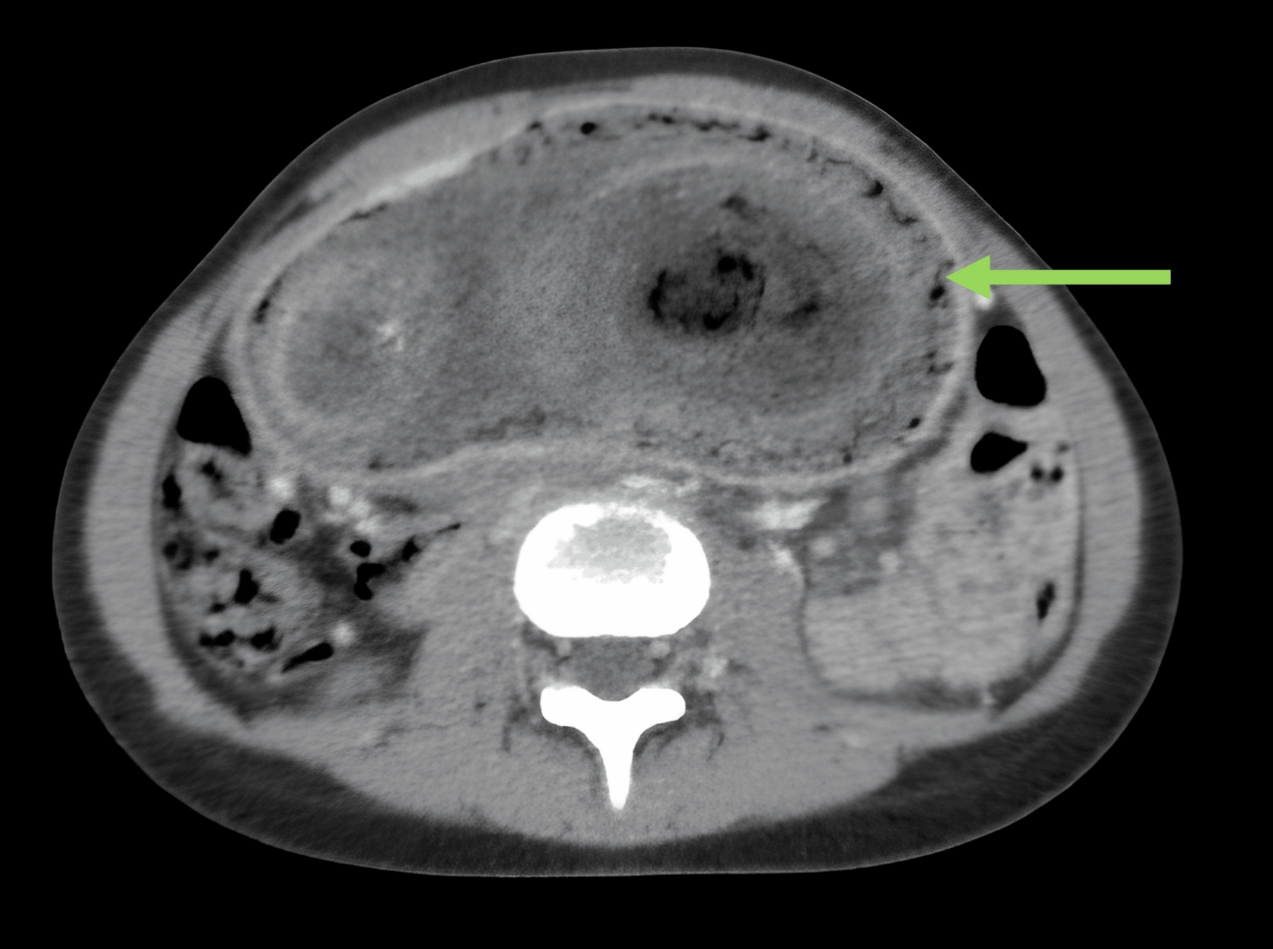
Abdominal CT ( transverse section) showing a thin wall enhanced after contrast injection (arrow).

A 16-year-old female, a high school student, presented with extreme fatigue, general weakness, weight loss of around 8 kg in four months, dyspepsia, and abdominal pain with a heaviness type evolving for three months. The patient reported regular menstrual cycles and revealed a history of trichotillomania and trichophagia during stressful episodes related to her schooling. General examination revealed pallor, with a body mass index (BMI) of 15 kg/m^2^. A physical examination revealed a firm, immobile, non-compressible, and painless mass extending from the epigastrium to the hypogastric region, measuring 20 cm along its major axis. The initial blood tests revealed severe iron deficiency anemia, with hemoglobin levels as low as 6 g/dL, and severe protein deficiency. Abdomino-pelvic CT scan demonstrated a diffuse, heterogeneous intragastric mass containing trapped air and food debris (Figure 1), with "compressed concentric rings" and a thin wall that showed enhancement after contrast administration (Figure 2); other abdominal organs appeared normal. These imaging findings led to a diagnosis of bezoar.

**Figure 3:**
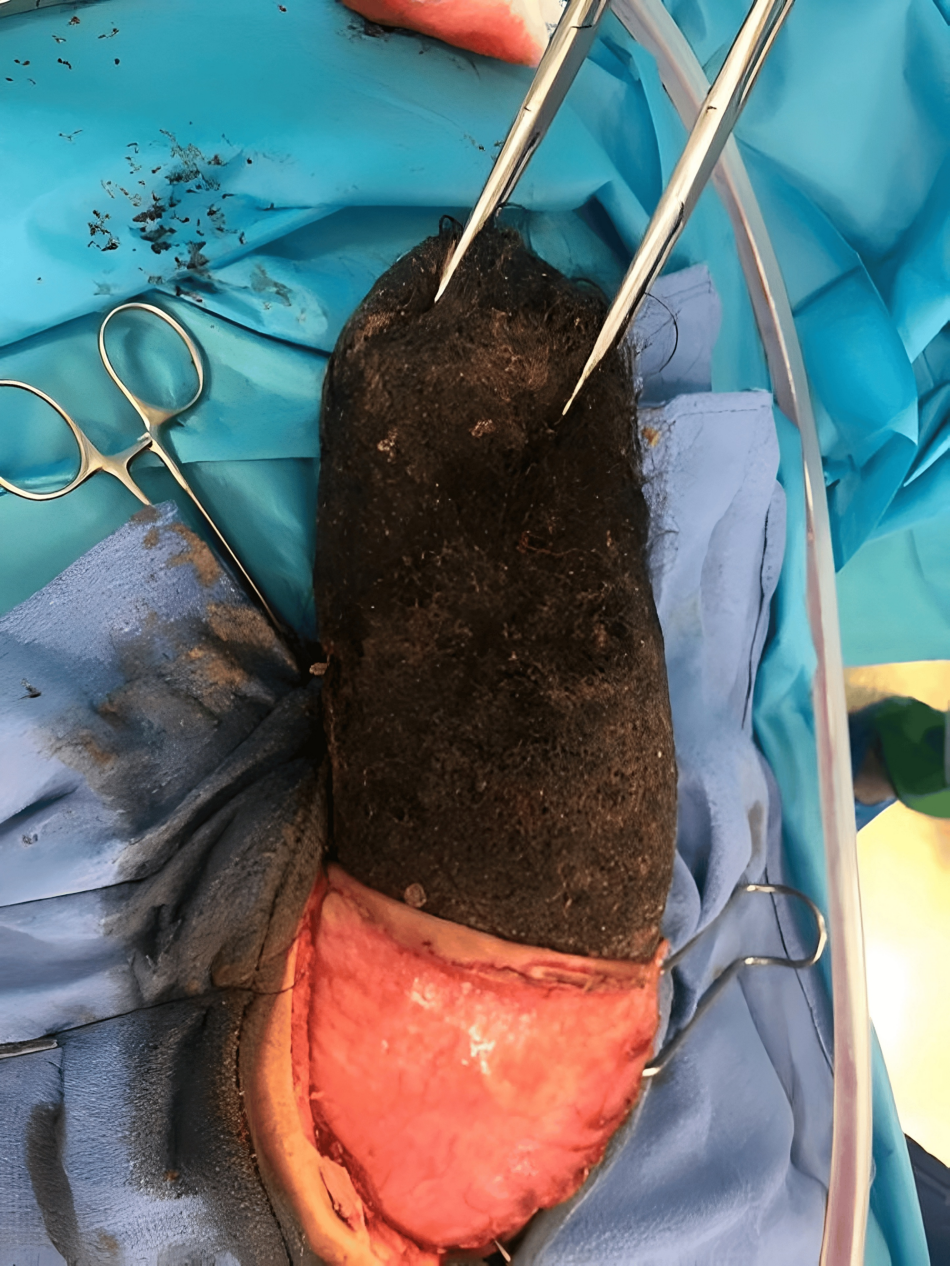
Intraoperative view showing the extraction of the trichobezoar

**Figure 4:**
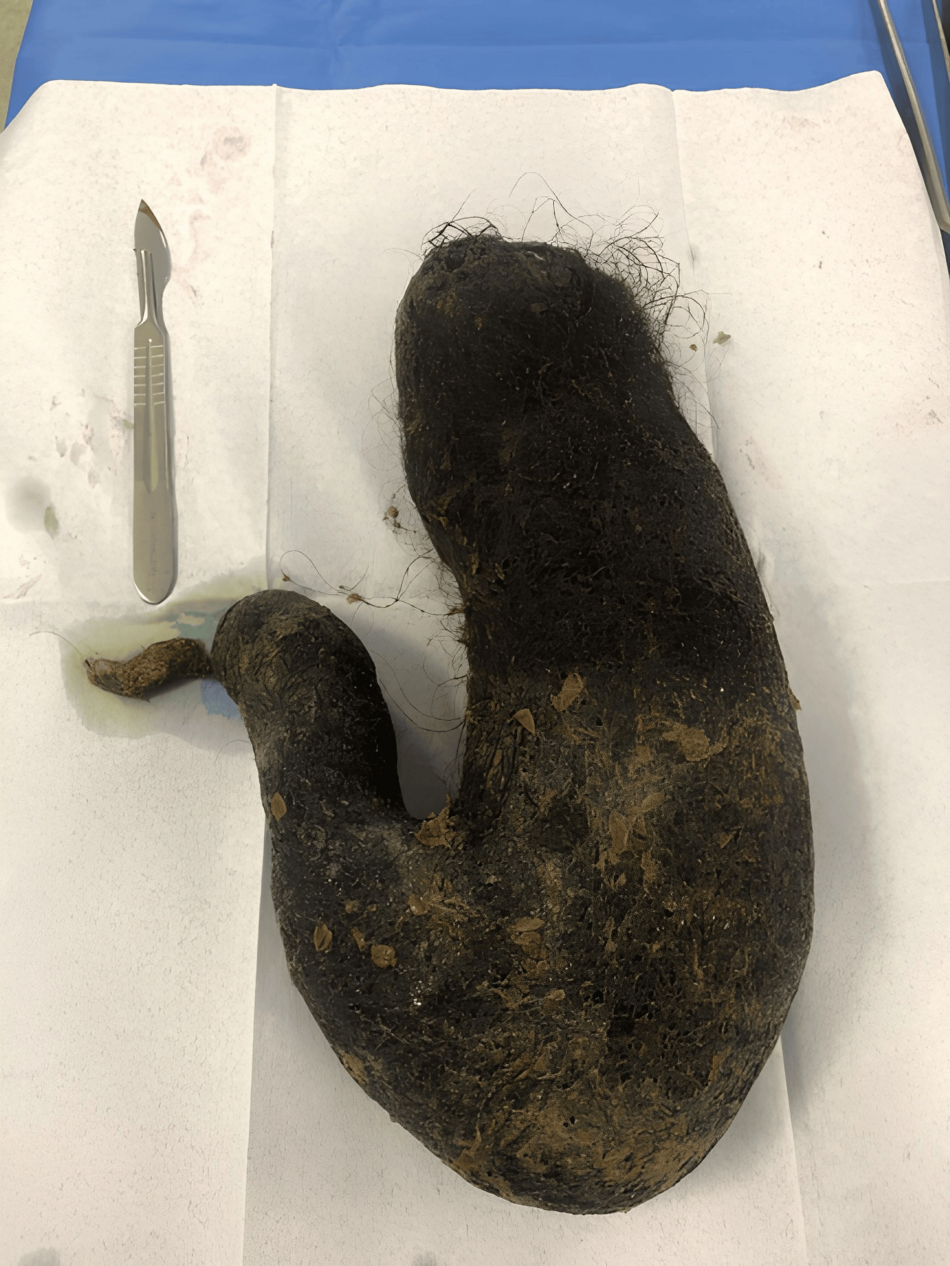
The trichobezoar after being extracted

The patient required preparation for the surgical procedure with transfusion of three whole blood bags, and she underwent laparotomic gastrotomy for trichobezoar removal. The procedure was successful, with no surgical complications (Figures 3, 4). The patient was discharged on the fifth postoperative day and referred to a psychiatrist for further care. After six months of postoperative follow-up, the patient showed no signs of recurrence, with a control hemoglobin level of 11g/dL and significant weight gain.

## Discussion

Trichobezoar is a rare condition predominantly affecting females (90% of cases), with 80% occurring in individuals under 30, peaking between 10 and 19 years of age [3]. Most trichobezoars are located in the stomach; however, if they extend through the pylorus into the jejunum, ileum, or colon, this is referred to as Rapunzel syndrome. Bezoars vary in composition, including lactobezoars (curdled milk, often in infants), phytobezoars (undigested plant matter), and trichobezoars, which constitute approximately 55% of all cases. Trichobezoars are composed of hair, fibers, and food debris and are typically confined to the stomach [4]. Trichobezoars may remain asymptomatic for long periods or present with symptoms such as epigastric discomfort (80%), abdominal pain (70%), nausea or vomiting (65%), asthenia with weight loss (38%), or gastrointestinal transit disorders (33%) such as diarrhea or constipation. A well-defined, hard, smooth, and movable abdominal mass in the epigastric area is frequently seen on clinical examination, along with possible symptoms of baldness or bad breath [5]. If a trichobezoar is left undetected or untreated, it can lead to harmful complications including failure to thrive, gastric ulceration and bleeding, gastric or duodenal perforation, upper gastrointestinal hemorrhage, peritonitis, pancreatitis, intussusception, biliary perforation, jaundice, and fistula formation [6]. In our case, anemia resulted from chronic blood loss, nutritional deficiencies, or malabsorption due to gastric obstruction, subsequently responsible for weight loss. Ultrasound allows visualization of a superficial, hyperechoic curvilinear band with a distinct posterior shadow in only 25% of cases [5]. The CT scan provides superior localization of the bezoar, showing well-defined intraluminal masses of low density containing air bubbles and leading to proximal intestinal dilation [7]. MRI may show variable appearances based on composition (air, water, food residues) [5]. However, CT with digestive tract opacification and MRI are less useful for diagnosing trichobezoars [4]. In our case, the CT scan results and the published clinical findings were sufficient to confirm the diagnosis and recommend surgery following a preoperative evaluation. Various treatments have been reported. For small trichobezoars, some authors recommend copious fluids combined with prokinetic agents, while others suggest endoscopic extraction. Other methods include fragmentation through laser or extracorporeal lithotripsy. However, these approaches can risk iatrogenic complications such as esophageal or intestinal occlusion from trichobezoar fragments. Therefore, surgical intervention is often necessary. Surgery allows exploration of the digestive tract, extraction of the gastric trichobezoar by gastrotomy, and removal of distal extensions or fragments by enterotomy. Recently, laparoscopy has emerged as a viable alternative to traditional laparotomy in select cases. Ongoing psychiatric care remains essential [8].

## Conclusions

Trichobezoar, a rare condition often associated with trichotillomania and trichophagia, can lead to significant gastrointestinal complications and systemic effects, including anemia. Anemia in such cases may result from chronic blood loss, nutritional deficiencies, or malabsorption due to gastrointestinal obstruction. The diagnosis is confirmed by esophagogastroduodenoscopy, while radiological imaging, particularly by CT scan, is crucial for identifying other locations and potential complications. Surgical intervention is the preferred treatment. However, the importance of comprehensive psychiatric care should not be overlooked, as it can be difficult to implement and accept in certain contexts.

## References

[1] Jalil S, Azhar M, ElKadi TTH (2023). Paediatric gastrointestinal trichobezoar—an uncommon entity: a case series with recent literature review. Ann Pediatr Surg.

[2] García-Ramírez BE, Nuño-Guzmán CM, Zaragoza-Carrillo RE, Salado-Rentería H, Gómez-Abarca A, Corona JL (2018). Small-bowel obstruction secondary to ileal trichobezoar in a patient with Rapunzel syndrome. Case Rep Gastroenterol.

[3] Lalith S, Gopalakrishnan KL, Ilangovan G, Jayajothi A (2017). Rapunzel syndrome. J Clin Diagn Res.

[4] Moujahid M, Ziadi T, Ennafae I, Kechna H, Ouzzad O, El Kandry S (2011). [A case of gastric trichobezoar]. Pan Afr Med J.

[5] Ousadden A, Mazaz K, Mellouki I, Taleb KA (2004). [Gastric trichobezoar: one case report] [Article in French]. Ann Chir.

[6] Lyons D (2019). Large gastric trichobezoar causing failure to thrive and iron deficiency anaemia in an adolescent girl: a case report emphasising the imaging findings and review of the literature. BJR Case Rep.

[7] Sulaiman Ambusaidi FM, Al-Yaqoubi M (2020). Gastric bezoar. Int J Pediatr Adolesc Med.

[8] Ezziti M, Haddad F, Tahiri M (2017). [Gastric trichobezoar: about a case] [Article in French]. Pan Afr Med J.

